# Impact of national commissioning of pre-exposure prophylaxis (PrEP) on equity of access in England: a PrEP-to-need ratio investigation

**DOI:** 10.1136/sextrans-2023-055989

**Published:** 2024-03-20

**Authors:** Flavien Coukan, Ann Sullivan, Holly Mitchell, Sajjida Jaffer, Andy Williams, John Saunders, Christina Atchison, Helen Ward

**Affiliations:** 1 National Institute for Health Research Applied Research Collaboration North West London, Chelsea and Westminster Hospital, London, UK; 2 Patient Experience Research Centre, School of Public Health, Imperial College London, London, UK; 3 Chelsea and Westminster Hospital NHS Foundation Trust, London, UK; 4 Imperial College London, London, UK; 5 Blood Safety, Hepatitis, Sexually Transmitted Infections (STI) and HIV Division, UK Health Security Agency, London, UK; 6 The Royal Marsden Hospital, London, UK; 7 The Royal London Hospital, London, UK; 8 UCL Centre for Clinical Research in Infection and Sexual Health, Institute for Global Health, University College London, London, UK; 9 National Institute for Health Research Imperial Biomedical Research Centre, London, UK

**Keywords:** HIV, Pre-Exposure Prophylaxis, Delivery of Health Care, National Health Programs

## Abstract

**Objectives:**

HIV pre-exposure prophylaxis (PrEP) is highly effective in preventing HIV acquisition. In England, NHS availability was limited to participants of the PrEP Impact Trial until late 2020. Some key populations at greater risk of HIV were under-represented in the trial suggesting inequities in trial PrEP access. We used the PrEP-to-need ratio (PnR; number of PrEP users divided by new HIV diagnoses) to investigate whether PrEP access improved following routine commissioning in October 2020 and identify populations most underserved by PrEP.

**Methods:**

Aggregated numbers of people receiving ≥1 PrEP prescription and non-late new HIV diagnoses (epidemiological proxy for PrEP need) were taken from national surveillance data sets. We calculated the PnR across socio-demographics during Impact (October 2017 to February 2020; pre-COVID-19 pandemic) and post-commissioning PrEP era (2021) in England.

**Results:**

PnR increased >11 fold, from 4.2 precommissioning to 48.9 in 2021, due to a fourfold reduction in non-late new HIV diagnoses and near threefold increase in PrEP users. PnR increased across genders, however, the men’s PnR increased 12-fold (from 5.4 precommissioning to 63.9 postcommissioning) while the women’s increased sevenfold (0.5 to 3.5). This increasing gender-based inequity was observed across age, ethnicity and region of residence: white men had the highest PnR, increasing >13 fold (7.1 to 96.0), while Black African women consistently had the lowest PnR, only increasing slightly (0.1 to 0.3) postcommissioning, suggesting they were the most underserved group. Precommissioning, the PnR was 78-fold higher among white men than Black women, increasing to 278-fold postcommissioning.

**Conclusions:**

Despite the overall increase in PrEP use, substantial PrEP Impact trial inequities widened postcommissioning in England, particularly across gender, ethnicity and region of residence. This study emphasises the need to guide HIV combination prevention based on equity metrics relative to the HIV epidemic. The PnR could support the optimisation of combination prevention to achieve zero new HIV infections in England by 2030.

**WHAT IS ALREADY KNOWN ON THIS TOPIC**

- In the United Kingdom (UK), men who have sex with men have championed HIV pre-exposure prophylaxis (PrEP) as part of comprehensive HIV combination prevention and are the majority of PrEP users across nations following its commissioning.

**WHAT THIS STUDY ADDS**

- This study shows that following PrEP commissioning in late 2020 in England, equity gaps across gender, age, ethnicity and region of residence widened significantly, as demonstrated by the PrEP-to-need ratio (PnR) equity metric; although this rose across all groups.

**HOW THIS STUDY MIGHT AFFECT RESEARCH, PRACTICE OR POLICY**

- The PnR is a pragmatic standardised metric to identify those most underserved by PrEP relative to the HIV epidemic. These populations will require specific attention via involvement and engagement to end HIV transmission by 2030 as they are also underserved by other HIV preventions and treatments.

## Introduction

The HIV epidemic has evolved substantially in the United Kingdom (UK) over the last two decades. The number of new HIV diagnoses peaked at just under 8000 in 2005, falling to 2955 in 2021, a 64% reduction.^1^ In England, this reduction was largely driven by a threefold drop in new HIV diagnoses among men who have sex with men (MSM) since mid-2010.^1^ This decline has not been as marked in other key populations such as Black women, people of Asian ethnicity, injecting drug users and those residing outside of London, partially explained by a differential uptake of HIV testing.^1^

HIV pre-exposure prophylaxis (PrEP) involves HIV-negative people taking antiretrovirals to prevent HIV acquisition and is highly effective in preventing infection.^2–4^ Wales and Scotland commissioned PrEP programmes in 2017, and Northern Ireland provided an extended pilot from 2018.^5^ However, in England, PrEP was initially only accessible via the Impact trial, which was limited to 26 000 places from 2017 until July 2020, when the Department of Health and Social Care announced routine PrEP commissioning. This commissioning was restricted to specialist sexual health services (SSHS); open access services which anyone can attend for free regardless of residency status.^6^ Findings from the PrEP Impact trial, delivered in SSHS, showed that 96% of participants were MSM, a population that accounted for <40% of all new HIV diagnoses in 2020.^1 7^ The Welsh and Scottish PrEP programmes have shown similar under-representation of non-MSM populations.^8 9^

A recent systematic review found many factors hindering access to PrEP in the UK, including lack of PrEP knowledge, lack of self-perception of HIV risk, HIV stigma and lack of access to a PrEP provider.^10^ It also highlighted MSM as the most studied population, reflecting the under-representation of other populations at high risk of HIV acquisition in PrEP use research.

The delivery of PrEP in England, therefore, needs to be more equitable, in line with the UK Government’s 2022 HIV Action Plan^11^: doing so requires a better understanding of which populations are most underserved by PrEP delivery. This will support further research into the specific barriers faced by these populations to identify potential ways to increase PrEP access.

### Objective

This study aimed to investigate whether equity of access to PrEP (as estimated by PrEP-to-need ratio (PnR)) improved following its commissioning at the end of the PrEP Impact trial. We hypothesised that the unlimited supply following PrEP commissioning would lead to an increase in equity of access across populations at risk.

## Methods

We performed a cross-sectional analysis of population-level data on PrEP use and non-late new HIV diagnoses to describe the distribution of the PnR during the precommissioning and postcommissioning era of PrEP.

### Data sources and definitions

#### Precommissioning versus postcommissioning era

The precommissioning era is defined as the first 29 months of the PrEP Impact trial (total 34 months), from October 2017 to end of February 2020. This represents the trial period prior to the COVID-19 pandemic, as the ensuing lockdowns resulted in a reduction in the provision of SSHS in the UK,^12^ which caused many individuals to pause or discontinue PrEP use.^13^ Meanwhile, the postcommissioning era was defined as the whole of 2021, the first full calendar year for which data were available following the commissioning of PrEP in October 2020.

#### PrEP use data

Aggregated data regarding the number of PrEP Impact trial participants up to February 2020 who received at least one PrEP prescription were provided by the UK Health Security Agency (UKHSA). This population was identified via linkage of the Impact trial electronic case report forms,^14^ and the GUMCAD STI surveillance system.^15^ GUMCAD is the English pseudoanonymised surveillance system that collects data on sexually transmitted infections (STI) tests, diagnoses and treatments, sexual behaviours, partner notification outcomes and PrEP provision.^15^

Aggregated data of the number of SSHS attendees who obtained PrEP on the NHS in 2021 were identified in the GUMCAD dataset (UKHSA), where clinic attendees with any of the following surveillance codes were included: PrEP uptake/continuation, PrEP regimen (daily or event-based) and/or PrEP prescription (30/60/90/180 tablets or other).^15^

#### New HIV diagnoses data

The aggregated numbers of new HIV diagnoses made between October 2017 and February 2020 and in 2021 were identified via the HIV and AIDS Reporting System (HARS; UKHSA) and restricted to residents in England.^16^ This was the best indicator of the epidemiological need for PrEP as the real-time incidence of HIV data is not available. HARS collects data on all new HIV diagnoses, first AIDS diagnoses and deaths from sexual health and HIV clinics, laboratories and other healthcare and community settings.^16^

As our interest was in acquisitions, which could reasonably have been expected to be prevented by PrEP, we excluded people who were diagnosed late based on an AIDS-defining illness at diagnosis, and the UK definition of a late diagnosis (a CD4 count of fewer than 350 cells/mm^3^ within 91 days of diagnosis, and no evidence of recent seroconversion).^16^ Those newly diagnosed in England with a prior diagnosis abroad were also excluded.

#### PrEP-to-need ratio

The PnR follows the methods developed by Siegler *et al* 17 and was calculated to investigate the population levels of PrEP use compared with the underlying epidemiological need for PrEP (via the non-late new HIV diagnosis proxy): we define it as the ratio of the number of PrEP users accessing SSHS to the number of non-late new HIV diagnoses for a given population. PnRs were calculated overall, by gender, by age and gender, by ethnicity and gender and by Index of Multiple Deprivation^18^ and gender at the national level; and by gender and ethnicity at the regional level. Gender was categorised into men and women, which included transgender men and women, respectively. Of note, the sexual orientation for men was only requested for the national gender breakdown to avoid small number masking for heterosexual men precommissioning, as required by the UKHSA data request policy.^19^ Due to UKHSA’s policy of small number masking, it was not possible to avoid the masking of aggregated numbers for certain sub-populations: in those instances, we used the highest number possible that could fit within the masking e.g. if the number of new HIV diagnoses was masked as ‘<5’, we used the number ‘4’ in the PnR calculations. Following evidence that people from Latin America have high HIV prevalence, a Latin American flag was derived from the country of birth of the attendees to investigate equity issues in that population, as this ethnic category is not available in the UK.^20^ Finally, Lorenz curves were used to display the disparities in PrEP uptake across ethnicity and gender nationally and by gender at the regional level: these graphical representations illustrate the distribution of PrEP users as a function of the cumulative percentage of HIV diagnoses. Data analysis was conducted in Stata V.17.0.

#### Significance testing

The equity gap was defined as the relative difference (ie, comparative ratio) between the PnR of the group of interest and the PnR of the baseline group. The relative differences and their respective p values and 95% CI were calculated via univariate logistic regression for the gender-only breakdown and bivariate logistic regressions for the others.

#### Sensitivity analyses

Sensitivity analyses were performed for the PnR approach, first replacing the denominator of the PnR with the number of SSHS attendees with a PrEP need identified.^21^ This was done only for the postcommissioning year of PrEP, as these data were not available in the GUMCAD data set for the Impact trial period. Second, we replaced the denominator with the number of new HIV diagnoses deemed recent by the Recent Infection Testing Algorithm (RITA), where small number masking allowed. RITA combines serological recency test results with clinical data to determine whether the diagnosis was of an HIV acquisition within 6 months.^16^

## Results

Between October 2017 and February 2020, 21 292 participants were recruited into the PrEP Impact Trial, and there were 5019 non-late new HIV diagnoses among England residents (table 1), which represented a PnR of 4.2; that is, there were just over four PrEP users for each non-late new HIV diagnosis. In 2021, there were nearly three times as many PrEP users (n=60 384), and a fourfold decrease in non-late new HIV diagnoses (n=1234), increasing the overall PnR to 48.9, an 11-fold increase from the precommissioning level (table 1).

**Table 1:** Distribution of the number of PrEP users and PrEP need in England during the precommissioning (PrEP Impact Trial—October 2017–February 2020) and postcommissioning (2021) period of PrEP by age and gender (including gender and sexual orientation minorities)

Age	Gender	Precommissioning (October 2017–February 2020)			Postcommissioning (2021)		
		PrEP users N (%)	Non-late new HIV dx N (%)	PnR	PrEP users N (%)	Non-late new HIV dx N (%)	PnR
Overall	Total	21 292 (100.0%)	5019 (100.0%)	4.2	60 384 (100.0%)	1234 (100.0%)	48.9
	Men, of which:	20 626 (96.9%)	3797 (75.7%)	5.4	57 169 (94.7%)	895 (72.5%)	63.9
	MSM	20 349 (95.6%)	2423 (48.3%)	8.4	49 543 (82.0%)	483 (39.1%)	102.6
	Heterosexual men	277 (1.3%)	1374 (27.4%)	0.2	7626 (12.6%)	412 (33.4%)	18.5
	Women	623 (2.9%)	1219 (24.3%)	0.5	1198 (2.0%)	338 (27.4%)	3.5
	Transgender people*	456 (2.1%)	9 (0.2%)	50.7	527 (0.9%)	4 (0.3%)	131.6
16–24	Men	2973 (14.0%)	538 (10.7%)	5.5	8691 (14.4%)	104 (8.4%)	83.6
	Women	115 (0.5%)	146 (2.9%)	0.8	261 (0.4%)	27 (2.2%)	9.7
	Subtotal	3104 (14.6%)	686 (13.7%)	4.5	9313 (15.4%)	132 (10.7%)	70.6
25–34	Men	8348 (39.2%)	1338 (26.7%)	6.2	23 603 (39.1%)	327 (26.5%)	72.2
	Women	251 (1.2%)	325 (6.5%)	0.8	492 (0.8%)	85 (6.9%)	5.8
	Subtotal	8617 (40.5%)	1664 (33.2%)	5.2	24 830 (41.1%)	412 (33.4%)	60.3
35–49	Men	6832 (32.1%)	1231 (24.5%)	5.5	17 972 (29.8%)	314 (25.4%)	57.2
	Women	182 (0.9%)	474 (9.4%)	0.4	348 (0.6%)	154 (12.5%)	2.3
	Subtotal	7021 (33.0%)	1705 (34.0%)	4.1	18 889 (31.3%)	468 (37.9%)	40.4
50–64	Men	2202 (10.3%)	579 (11.5%)	3.8	6184 (10.2%)	119 (9.6%)	52.0
	Women	64 (0.3%)	226 (4.5%)	0.3	88 (0.1%)	65 (5.3%)	1.4
	Subtotal	2267 (10.6%)	805 (16.0%)	2.8	6583 (10.9%)	184 (14.9%)	35.8
65 and over	Men	271 (1.3%)	111 (2.2%)	2.4	718 (1.2%)	31 (2.5%)	23.2
	Women	11 (0.1%)	48 (1.0%)	0.2	7 (0.0%)	7 (0.6%)	1.0
	Subtotal	283 (1.3%)	159 (3.2%)	1.8	766 (1.3%)	38 (3.1%)	20.2

* Transgender people include transgender men, transgender women and those who identified as non-binary and were only available for the national gender breakdown to avoid small number masking, as required by UKHSA data request policy.

MSM, men who have sex with men; PnR, PrEP-to-need ratio; PrEP, pre-exposure prophylaxis; UKHSA, UK Health Security Agency.

Almost all PrEP Impact Trial participants were men (96.9%), of whom most were MSM (98.7%). The overall proportion of PrEP users who were MSM declined from 95.6% to 82.0% from pre to postcommissioning (table 1). This was due to an increase in the proportion of heterosexual men PrEP users (it increased from 1.3% to 12.6%). This contrasts with women accounting for a lower share of PrEP users (from 2.9% to 2.0%) despite accounting for a higher proportion of non-late new HIV diagnoses in 2021 (n=338, 27.4%) than previous (n=1219, 24.3%). This resulted in the equity gap between women and men widening by nearly 70% as the men’s PnR was 11 times greater than women’s during the Impact Trial but increased to 16 times greater postcommissioning (online supplemental material 1). The widening inequity between men and women was found across all age categories, ethnicities and regions of residence.

10.1136/sextrans-2023-055989.supp1 Supplementary data

White men had the highest PnR both pre and postcommissioning at 7.1 and 96.0, respectively (table 2), while Black African women had the lowest at 0.1 and 0.3, making them the most underserved group in relation to PrEP in England both pre and postcommissioning. The equity gap between these groups increased from a 78-fold difference precommissioning to a 278-fold difference postcommissioning (online supplemental material 1), a 3.6-fold increase in the equity gap between white men and Black African women across time periods (relative difference=0.28 (95% CI 0.17 to 0.46)). This racial divide held true across regions of residence, whereby Black Africans consistently had the lowest PnR, and those of white ethnicity consistently had the highest (online supplemental material 1). People of Black Caribbean and ‘Black other’ ethnicity also consistently had the second and third-lowest PnR.

**Table 2:** Distribution of the number of PrEP users and PrEP need in England during the precommissioning (PrEP Impact Trial—October 2017–February 2020) and postcommissioning (2021) period of PrEP by ethnicity and gender

Ethnicity	Gender	Precommissioning (October 2017–February 2020)			Postcommissioning (2021)		
		PrEP users N (%)	Non-late new HIV dx N (%)	PnR	PrEP users N (%)	Non-late new HIV dx N (%)	PnR
White	Men	15 669 (73.6%)	2196 (43.8%)	7.1	41 765 (69.2%)	435 (35.3%)	96.0
	Women	367 (1.7%)	367 (7.3%)	1.0	714 (1.2%)	90 (7.3%)	7.9
	Subtotal	16 061 (75.4%)	2564 (51.1%)	6.3	44 106 (73.0%)	526 (42.6%)	83.9
Black African	Men	339 (1.6%)	293 (5.8%)	1.2	1050 (1.7%)	79 (6.4%)	13.3
	Women	36 (0.2%)	395 (7.9%)	0.1	42 (0.1%)	122 (9.9%)	0.3
	Subtotal	376 (1.8%)	688 (13.7%)	0.5	1131 (1.9%)	201 (16.3%)	5.6
Black Caribbean	Men	341 (1.6%)	86 (1.7%)	4.0	964 (1.6%)	28 (2.3%)	34.4
	Women	9 (0.0%)	38 (0.8%)	0.2	19 (0.0%)	15 (1.2%)	1.3
	Subtotal	350 (1.6%)	124 (2.5%)	2.8	996 (1.6%)	43 (3.5%)	23.2
Black Other	Men	128 (0.6%)	66 (1.3%)	1.9	336 (0.6%)	22 (1.8%)	15.3
	Women	6 (0.0%)	43 (0.9%)	0.1	13 (0.0%)	7 (0.6%)	1.9
	Subtotal	134 (0.6%)	109 (2.2%)	1.2	358 (0.6%)	29 (2.4%)	12.3
Asian	Men	1041 (4.9%)	240 (4.8%)	4.3	4413 (7.3%)	69 (5.6%)	64.0
	Women	46 (0.2%)	51 (1.0%)	0.9	86 (0.1%)	13 (1.1%)	6.6
	Subtotal	1092 (5.1%)	292 (5.8%)	3.7	4616 (7.6%)	82 (6.6%)	56.3
Mixed/other	Men	1646 (7.7%)	411 (8.2%)	4.0	4675 (7.7%)	76 (6.2%)	61.5
	Women	87 (0.4%)	85 (1.7%)	1.0	175 (0.3%)	25 (2.0%)	7.0
	Subtotal	1740 (8.2%)	497 (9.9%)	3.5	4960 (8.2%)	101 (8.2%)	49.1
Not stated	Men	1462 (6.9%)	505 (10.1%)	2.9	3966 (6.6%)	186 (15.1%)	21.3
	Women	72 (0.3%)	240 (4.8%)	0.3	149 (0.2%)	66 (5.3%)	2.3
	Subtotal	1539 (7.2%)	745 (14.8%)	2.1	4217 (7.0%)	252 (20.4%)	16.7
Latin American*	Men	714 (3.4%)	216 (4.3%)	3.3	2459 (4.1%)	49 (4.0%)	50.2
	Women	81 (0.4%)	21 (0.4%)	3.9	218 (0.4%)	4 (0.3%)	54.5
	Subtotal	796 (3.7%)	238 (4.7%)	3.3	2702 (4.5%)	52 (4.2%)	52.0

*Latin American is not an ethnicity readily available in the GUMCAD STI surveillance system and HARS datasets and was derived from the attendee’s country of birth (Belize, Costa Rica, El Salvador, Guatemala, Honduras, Mexico, Nicaragua, Panama, Argentina, Bolivia, Bouvet, Brazil, Chile, Colombia, Ecuador, Falkland Islands, French Guiana, Guyana, Paraguay, Peru, South Georgia and the South Sandwich Islands, Suriname, Uruguay, Venezuela)

HARS, HIV and AIDS Reporting System; PnR, PrEP-to-need ratio; PrEP, pre-exposure prophylaxis.

**Figure 1:**
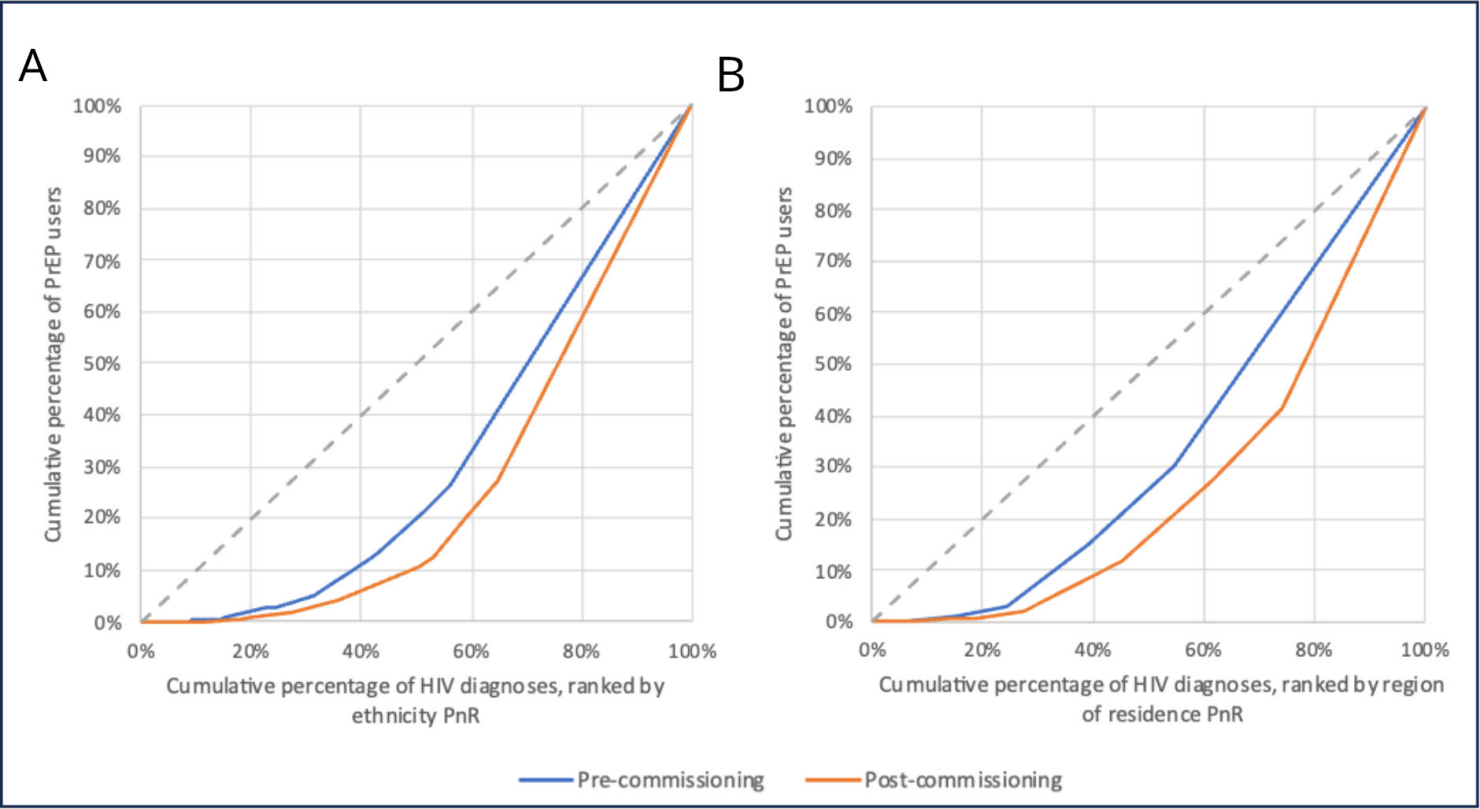
Lorenz curve (Latin American is not an ethnicity readily available in the GUMCAD STI surveillance system and HARS datasets and was derived from the attendee’s country of birth (Belize, Costa Rica, El Salvador, Guatemala, Honduras, Mexico, Nicaragua, Panama, Argentina, Bolivia, Bouvet, Brazil, Chile, Colombia, Ecuador, Falkland Islands, French Guiana, Guyana, Paraguay, Peru, South Georgia and the South Sandwich Islands, Suriname, Uruguay, Venezuela)) of the cumulative proportion of PrEP users by the cumulative proportion of non-late new HIV diagnoses by (A) ethnicity and gender grouping and (B) region of residence and gender grouping in England precommissioning (blue curve) and postcommissioning (orange curve). HARS, HIV and AIDS Reporting SystemPrEP, pre-exposure prophylaxis.

The Lorenz curves, figure 1A, highlight the widening inequity: precommissioning, low PnR ethnicity and gender groupings, who made up 20% of PrEP users, accounted for 50% of non-late new HIV diagnoses, which increased to nearly 60% postcommissioning.

Precommissioning, while men from London accounted for one-third of all non-late new HIV diagnoses, they made up over half all PrEP users (table 3, figure 1B), which changed to 55.2% of all PrEP users and a quarter of HIV diagnoses in 2021. This represents a 16-fold increase in the PnR among men resident in London pre to postcommissioning (relative difference=16.06 (95% CI 14.22 to 18.14)), the highest across regions of residence and gender groupings (online supplemental material 1). In comparison, residents in the Midlands and East of England have the lowest PnR across time periods, and the lowest PnR increase: the equity gap compared with men residing in London increased twofold (relative difference=0.49 (95% CI 0.40 to 0.59)) and nearly threefold (relative difference=0.36 (95% CI 0.25 to 0.52)) for the men and women residing in that region, respectively (online supplemental material 1).

**Table 3:** Distribution of the number of PrEP users and PrEP need in England during the precommissioning (PrEP Impact Trial—October 2017–February 2020) and postcommissioning (2021) period of PrEP by region of residence and gender

Region	Gender	Precommissioning (October 2017–February 2020)			Postcommissioning (2021)		
		PrEP users N (%)	Non-late new HIV dx N (%)	PnR	PrEP users N (%)	Non-late new HIV dx N (%)	PnR
London	Men	10 927 (52.1%)	1706 (34.0%)	6.4	32 708 (55.2%)	318 (25.8%)	102.9
	Women	330 (1.6%)	454 (9.0%)	0.7	668 (1.1%)	108 (8.8%)	6.2
	Subtotal	11 275 (53.8%)	2161 (43.1%)	5.2	33 569 (56.6%)	426 (34.5%)	78.8
Midlands & East of England	Men	2518 (12.0%)	737 (14.7%)	3.4	5816 (9.8%)	218 (17.7%)	26.7
	Women	92 (0.4%)	334 (6.7%)	0.3	152 (0.3%)	96 (7.8%)	1.6
	Subtotal	2615 (12.5%)	1072 (21.4%)	2.4	6726 (11.3%)	314 (25.4%)	21.4
North of England	Men	3274 (15.6%)	783 (15.6%)	4.2	9231 (15.6%)	207 (16.8%)	44.6
	Women	84 (0.4%)	233 (4.6%)	0.4	180 (0.3%)	78 (6.3%)	2.3
	Subtotal	3366 (16.1%)	1016 (20.2%)	3.3	9858 (16.6%)	285 (23.1%)	34.6
South of England	Men	3595 (17.2%)	571 (11.4%)	6.3	8454 (14.3%)	152 (12.3%)	55.6
	Women	94 (0.4%)	198 (3.9%)	0.5	179 (0.3%)	56 (4.5%)	3.2
	Subtotal	3700 (17.7%)	770 (15.3%)	4.8	9147 (15.4%)	209 (16.9%)	43.8

PnR, PrEP-to-need ratio; PrEP, pre-exposure prophylaxis.

## Discussion

This is the first study to investigate inequity in PrEP use in England by comparing the number of PrEP users to the number of individuals who may have benefited from PrEP (using non-late new HIV diagnosis proxy) to identify the populations most underserved in relation to PrEP. We measured how PrEP inequity changed following the roll-out of a nationally commissioned routine PrEP service.

This ecological analysis highlighted large inequities across gender, age, ethnicity and region of residence. The PnR was significantly higher in men than women, in younger men than older men and women, in white men than minority ethnicity men and women and in London than in other parts of England. Additionally, these equity gaps widened significantly following PrEP commissioning: the PnR difference between men and women increased from an 11-fold to a 16-fold difference. This held true across age, ethnicity and region.

However, the most substantial equity gaps were at the intersection between ethnicity and gender. Black African women represented <1% all PrEP users before and after commissioning. However, they made up an increasing share of non-late new HIV diagnoses postcommissioning (from 7.9% to 9.9%). In contrast, while the proportion of white men among all PrEP users decreased slightly from 73.6% to 69.2%, the proportion of non-late new HIV diagnoses attributed to them reduced by 20%. This resulted in an equity gap widening substantially from a 78-fold difference precommissioning to a 278-fold difference postcommissioning. Furthermore, the under-representation of people of Black ethnicity was seen across all English regions.

### Limitations

This analysis has several limitations. First, missing data on ethnicity remained stable at 7% of the number of PrEP users but reached 20.4% of the number of non-late new HIV diagnoses postcommissioning from 14.8% precommissioning, that is, the proportion of missingness in the denominator was two times to three times that of the numerator. This could be due to differential missingness bias, overestimating the PnR estimates for some ethnicities while underestimating the estimates for others. The lack of more granular ethnicity categories could mask additional inequities among populations whose ethnicity identity is not available in the UK census; hence the interest to flag those born in Latin America, who have a PnR much lower than their white counterparts despite known high HIV prevalence in England.^20^

Second, this ecological study uses non-late new HIV diagnoses as a proxy for PrEP need. Those new diagnoses do not necessarily represent individuals with the highest risk for HIV acquisition, hence why our approach aimed to remove those diagnosed late in their HIV progression. We explored the use of new HIV diagnoses deemed recent by the RITA algorithm in the denominator of the PnR calculations as a sensitivity analysis (online supplemental material 2), but this is an inaccurate measure as RITA results are available for <50% of new diagnoses.

10.1136/sextrans-2023-055989.supp2 Supplementary data

We also performed a sensitivity analysis using GUMCAD PrEP need data, which confirmed the major equity gaps described here (online supplemental material 3). However, this was only available for 2021. Additionally, while the current methodology is imperfect in identifying those in need of PrEP,^22 23^ it remains the best proxy available: the third national Survey of Sexual attitudes and lifestyles showed that most people having condomless sex with new partner(s) do not attend SSHS^24^ and would, therefore, not be captured within the GUMCAD PrEP need data.

10.1136/sextrans-2023-055989.supp3 Supplementary data

Another limitation is that PrEP Impact trial data underestimate PrEP use (and, therefore, an underestimate of the PnR) over the duration of the trial. This is because demand from attendees outpaced the number of Impact places available, resulting in private purchase of PrEP,^25^ and, therefore, would not be included in the precommissioning numbers presented here. This is a problem most likely affecting MSM estimates, as they made the bulk of self-sourcing PrEP users,^26^ which would imply an underestimation of the equity gaps with other populations during Impact. However, our results showed equity issues similar to those reported in the Scottish and Welsh commissioned PrEP programmes.^8 9^

### Implications

By highlighting equity gaps in SSHS PrEP delivery, this study is particularly timely as it provides data to inform the UK Government aims to end new HIV transmission by the end of the decade in England.^11^

This equity analysis is helpful in identifying where equity gaps are to support those most underserved. The PnR methodology is a pragmatic standardised method that can be used to compare equity across UK nations and across other countries worldwide.^27^ While few other studies have used this method to date, they all showed the practicality of PnR calculations to highlight PrEP equity gaps; in fact, these studies (done in the USA) showed similar sociodemographics PrEP equity patterns to this analysis.^17 28^ They found that women, Black and older people, along with those from historically disadvantaged backgrounds, were the most underserved populations.

It is also important to note that while PrEP is not the only prevention available in England (PrEP is part of the wider HIV combination prevention package), the inequities highlighted in this paper mirror those affecting other HIV preventions: we know that women and people of Black African ethnicity have yet to recover pre-COVID-19 levels of HIV testing.^1^ This lower-than-expected HIV testing implies that these populations’ PnR was overestimated compared with other populations for whom HIV testing recovered to pre-COVID-19 levels, that is, the true equity gap with white men could be bigger than estimated here. Additionally, Black women and those residing outside of London living with HIV from the same populations are also known to experience unequal linkage to care and treatment and worse outcomes than their white cisgender counterparts.^1 29^

It is important to emphasise that PnR is an equity measure only as an ecological construct, that is, it explores disparities in PrEP access across sociodemographic variables at population level.^17^ In the future, it will be key to look at MSM and non-MSM populations separately when updating this analysis to better understand who within the MSM population requires more support. Similar analyses should be done for the other underserved populations highlighted in this paper (women and ethnic minorities), ideally at attendee level, to investigate why PrEP assessments and use were so low in these populations.^1^

Finally, addressing these equity gaps will also require all relevant stakeholders involved in the delivery of PrEP in England (Local Authority commissioners, clinicians and other healthcare workers, as well as those from the populations this analysis highlighted as underserved) to come together to decide on the best way forward to address these equity issues. Such work will be reliant on a better understanding of the various behavioural factors involved along that PrEP care continuum^30^ across the various underserved populations as different populations may struggle with different parts of care continuum engagement.

## Conclusions

PrEP delivery should be guided based on equity metrics (PrEP use relative to HIV epidemic), not PrEP equality (equal across groups, regardless of the proportion of HIV diagnoses). On this basis, the PrEP Impact trial and subsequent PrEP commissioned service demonstrate large and widening equity gaps by gender, ethnicity and geography, especially those of older age, women of Black ethnicity and those outside of London.

The PnR proved its efficacy in identifying the populations most underserved by the current PrEP service in England. To reach the UK Government’s aim to stop HIV transmissions by 2030,^11^ it will be key to address the barriers to PrEP access and other HIV preventions faced by these underserved populations.

## Data Availability

All data relevant to the study are included in the article or uploaded as supplementary information.
