# Supplementary material for: Impact of national commissioning of pre-exposure prophylaxis (PrEP) on equity of access in England: a PrEP-to-need ratio investigation
Source: Sex Transm Infect. 2024 Mar 20;100(3):166–72. doi: 10.1136/sextrans-2023-055989 (PMC11041607; doi:10.1136/sextrans-2023-055989)
Supplement: Supplementary data [file sextrans-2023-055989supp001.pdf]

BMJ Publishing Group Limited (BMJ) disclaims all liability and responsibility arising from any reliance placed on this supplemental material which has been supplied by the author(s)

Supplementary material 1

Table 1. Distribution of the number of PrEP users and PrEP need in England during the pre- (PrEP Impact Trial – October 2017 to February 2020) and post-commissioning (2021) period of PrEP by age and gender (including transgender and sexual orientation minorities)

Age	Gender	Pre-commissioning (Oct 2017 – Feb 2020)				Post-commissioning (2021)				Pre- to post-commissioning comparisons within groups	Pre- to post-commissioning comparisons across groups
		PrEP users N (%)	Non-late new HIV dx N (%)	PnR	PnR relative difference (95% CI)	PrEP users N (%)	Non-late new HIV dx N (%)	PnR	PnR relative difference (95% CI)	PnR relative difference (95% CI)	PnR relative difference (95% CI)
Overall	**Total**	**21,292 (100.0%)**	**5,019 (100.0%)**	**4.2**	**N/A**	**60,384 (100.0%)**	**1,234 (100.0%)**	**48.9**	**N/A**	**11.53* (10.82-12.30)**	**N/A**
	Men, of which:	20,626 (96.9%)	3,797 (75.7%)	5.4	Baseline group	57,169 (94.7%)	895 (72.5%)	63.9	Baseline group	11.76* (10.91-12.67)	Baseline group
	MSM	20,349 (95.6%)	2,423 (48.3%)	8.4	N/A	49,543 (82.0%)	483 (39.1%)	102.6	N/A	12.21* (11.06-13.48)	N/A
	Heterosexual men	277 (1.3%)	1,374 (27.4%)	0.2	N/A	7,626 (12.6%)	412 (33.4%)	18.5	N/A	91.81* (78.02-108.04)	N/A
	Women	623 (2.9%)	1,219 (24.3%)	0.5	0.09* (0.08-0.10)	1,198 (2.0%)	338 (27.4%)	3.5	0.06* (0.05-0.06)	6.94* (5.94-8.09)	0.59* (0.50-0.70)
	Transgender people[1]	456 (2.1%)	9 (0.2%)	50.7	N/A	527 (0.9%)	4 (0.3%)	131.6	N/A	2.60 (0.80-8.50)	N/A
16 to 24	Men	2,973 (14.0%)	538 (10.7%)	5.5	Baseline group	8,691 (14.4%)	104 (8.4%)	83.6	Baseline group	15.12* (12.21-18.73)	Baseline group
	Women	115 (0.5%)	146 (2.9%)	0.8	0.14* (0.11-0.19)	261 (0.4%)	27 (2.2%)	9.7	0.12* (0.07-0.18)	12.27* (7.70-19.55)	0.81 (0.49-1.35)
	**Subtotal**	**3,104 (14.6%)**	**686 (13.7%)**	**4.5**	**N/A**	**9,313 (15.4%)**	**132 (10.7%)**	**70.6**	**N/A**	**15.59* (12.89-18.87)**	**N/A**
25 to 34	Men	8,348 (39.2%)	1,338 (26.7%)	6.2	1.13** (1.01-1.26)	23,603 (39.1%)	327 (26.5%)	72.2	0.86 (0.69-1.08)	11.57* (10.23-13.09)	0.77** (0.60-0.98)
	Women	251 (1.2%)	325 (6.5%)	0.8	0.14* (0.12-0.17)	492 (0.8%)	85 (6.9%)	5.8	0.07* (0.05-0.09)	7.49* (5.65-9.95)	0.50* (0.35-0.71)
	**Subtotal**	**8,617 (40.5%)**	**1,664 (33.2%)**	**5.2**	**N/A**	**24,830 (41.1%)**	**412 (33.4%)**	**60.3**	**N/A**	**11.64* (10.42-13.00)**	**N/A**
35 to 49	Men	6,832 (32.1%)	1,231 (24.5%)	5.5	1.00 (0.90-1.12)	17,972 (29.8%)	314 (25.4%)	57.2	0.68* (0.55-0.86)	10.31* (9.08-11.71)	0.68* (0.53-0.87)
	Women	182 (0.9%)	474 (9.4%)	0.4	0.07* (0.06-0.08)	348 (0.6%)	154 (12.5%)	2.3	0.03* (0.02-0.04)	5.89* (4.56-7.60)	0.39* (0.28-0.54)
	**Subtotal**	**7,021 (33.0%)**	**1,705 (34.0%)**	**4.1**	**N/A**	**18,889 (31.3%)**	**468 (37.9%)**	**40.4**	**N/A**	**9.80* (8.82-10.90)**	**N/A**
50 to 64	Men	2,202 (10.3%)	579 (11.5%)	3.8	0.69* (0.60-0.78)	6,184 (10.2%)	119 (9.6%)	52.0	0.62* (0.48-0.81)	13.66* (11.15-16.74)	0.90 (0.67-1.21)
	Women	64 (0.3%)	226 (4.5%)	0.3	0.05* (0.04-0.07)	88 (0.1%)	65 (5.3%)	1.4	0.02* (0.01-0.02)	4.78* (3.13-7.31)	0.32* (0.20-0.51)
	**Subtotal**	**2,267 (10.6%)**	**805 (16.0%)**	**2.8**	**N/A**	**6,583 (10.9%)**	**184 (14.9%)**	**35.8**	**N/A**	**12.70* (10.75-15.02)**	**N/A**
65 and over	Men	271 (1.3%)	111 (2.2%)	2.4	0.44* (0.35-0.56)	718 (1.2%)	31 (2.5%)	23.2	0.28* (0.18-0.42)	9.49* (6.22-14.47)	0.63** (0.39-1.01)
	Women	11 (0.1%)	48 (1.0%)	0.2	0.04* (0.02-0.08)	7 (0.0%)	7 (0.6%)	1.0	0.01* (0.00-0.03)	4.36** (1.27-15.01)	0.29** (0.08-1.01)
	**Subtotal**	**283 (1.3%)**	**159 (3.2%)**	**1.8**	**N/A**	**766 (1.3%)**	**38 (3.1%)**	**20.2**	**N/A**	**11.33* (7.75-16.55)**	**N/A**

[1] Transgender people includes transgender men, transgender women and those who identified as non-binary and was only available for the national gender breakdown to avoid small number masking, as required by UKHSA data request policy.

* p-value<0.01

**p-value<0.1

Table 2. Distribution of the number of PrEP users and PrEP need in England during the pre- (PrEP Impact Trial – October 2017 to February 2020) and post-commissioning (2021) period of PrEP by ethnicity and gender

Ethnicity	Gender	Pre-commissioning (Oct 2017 – Feb 2020)				Post-commissioning (2021)				Pre- to post-commissioning comparisons within groups	Pre- to post-commissioning comparisons across groups
		PrEP users N (%)	Non-late new HIV dx N (%)	PnR	PnR relative difference (95% CI)	PrEP users N (%)	Non-late new HIV dx N (%)	PnR	PnR relative difference (95% CI)	PnR relative difference (95% CI)	PnR relative difference (95% CI)
White	Men	15,669 (73.6%)	2,196 (43.8%)	7.1	Baseline group	41,765 (69.2%)	435 (35.3%)	96.0	Baseline group	13.46* (12.12-14.94)	Baseline group
	Women	367 (1.7%)	367 (7.3%)	1.0	0.14* (0.12-0.16)	714 (1.2%)	90 (7.3%)	7.9	0.08* (0.07-0.10)	7.93* (6.10-10.32)	0.59* (0.44-0.78)
	**Subtotal**	**16,061 (75.4%)**	**2,564 (51.1%)**	**6.3**	**N/A**	**44,106 (73.0%)**	**526 (42.6%)**	**83.9**	**N/A**	**13.39* (12.17-14.73)**	**N/A**
Black African	Men	339 (1.6%)	293 (5.8%)	1.2	0.16* (0.14-0.19)	1,050 (1.7%)	79 (6.4%)	13.3	0.14* (0.11-0.18)	11.49* (8.71-15.15)	0.85 (0.63-1.15)
	Women	36 (0.2%)	395 (7.9%)	0.1	0.01* (0.01-0.02)	42 (0.1%)	122 (9.9%)	0.3	0.00* (0.00-0.01)	3.78* (2.32-6.16)	0.28* (0.17-0.46)
	**Subtotal**	**376 (1.8%)**	**688 (13.7%)**	**0.5**	**N/A**	**1,131 (1.9%)**	**201 (16.3%)**	**5.6**	**N/A**	**10.30* (8.47-12.52)**	**N/A**
Black Caribbean	Men	341 (1.6%)	86 (1.7%)	4.0	0.56* (0.44-0.71)	964 (1.6%)	28 (2.3%)	34.4	0.36* (0.24-0.53)	8.68* (5.57-13.54)	0.65** (0.41-1.02)
	Women	9 (0.0%)	38 (0.8%)	0.2	0.03* (0.02-0.07)	19 (0.0%)	15 (1.2%)	1.3	0.01* (0.01-0.03)	5.35* (1.98-14.44)	0.40** (0.15-1.08)
	**Subtotal**	**350 (1.6%)**	**124 (2.5%)**	**2.8**	**N/A**	**996 (1.6%)**	**43 (3.5%)**	**23.2**	**N/A**	**8.21* (5.68-11.85)**	**N/A**
Black Other	Men	128 (0.6%)	66 (1.3%)	1.9	0.27* (0.20-0.37)	336 (0.6%)	22 (1.8%)	15.3	0.16* (0.10-0.25)	7.88* (4.66-13.30)	0.59** (0.34-1.00)
	Women	6 (0.0%)	43 (0.9%)	0.1	0.02* (0.01-0.05)	13 (0.0%)	7 (0.6%)	1.9	0.02* (0.01-0.05)	13.31* (3.80-46.67)	0.99 (0.28-3.48)
	**Subtotal**	**134 (0.6%)**	**109 (2.2%)**	**1.2**	**N/A**	**358 (0.6%)**	**29 (2.4%)**	**12.3**	**N/A**	**10.04* (6.37-15.83)**	**N/A**
Asian	Men	1,041 (4.9%)	240 (4.8%)	4.3	0.61* (0.52-0.70)	4,413 (7.3%)	69 (5.6%)	64.0	0.67* (0.52-0.86)	14.75* (11.19-19.43)	1.10 (0.82-1.47)
	Women	46 (0.2%)	51 (1.0%)	0.9	0.13* (0.08-0.19)	86 (0.1%)	13 (1.1%)	6.6	0.07* (0.04-0.12)	7.33* (3.62-14.86)	0.55** (0.27-1.11)
	**Subtotal**	**1,092 (5.1%)**	**292 (5.8%)**	**3.7**	**N/A**	**4,616 (7.6%)**	**82 (6.6%)**	**56.3**	**N/A**	**15.05* (11.68-19.40)**	**N/A**
Mixed/ other	Men	1,646 (7.7%)	411 (8.2%)	4.0	0.56* (0.50-0.63)	4,675 (7.7%)	76 (6.2%)	61.5	0.64* (0.50-0.82)	15.36* (11.95-19.74)	1.14 (0.87-1.50)
	Women	87 (0.4%)	85 (1.7%)	1.0	0.14* (0.11-0.19)	175 (0.3%)	25 (2.0%)	7.0	0.07* (0.05-0.11)	6.84* (4.09-11.44)	0.51** (0.30-0.86)
	**Subtotal**	**1,740 (8.2%)**	**497 (9.9%)**	**3.5**	**N/A**	**4,960 (8.2%)**	**101 (8.2%)**	**49.1**	**N/A**	**14.03* (11.25-17.49)**	**N/A**
Not stated	Men	1,462 (6.9%)	505 (10.1%)	2.9	0.41* (0.36-0.45)	3,966 (6.6%)	186 (15.1%)	21.3	0.22* (0.19-0.26)	7.37* (6.16-8.80)	0.55* (0.45-0.67)
	Women	72 (0.3%)	240 (4.8%)	0.3	0.04* (0.03-0.05)	149 (0.2%)	66 (5.3%)	2.3	0.02* (0.02-0.03)	7.53* (5.09-11.13)	0.56* (0.37-0.84)
	**Subtotal**	**1,539 (7.2%)**	**745 (14.8%)**	**2.1**	**N/A**	**4,217 (7.0%)**	**252 (20.4%)**	**16.7**	**N/A**	**8.10* (6.94-9.45)**	**N/A**
Latin American[2]	Men	714 (3.4%)	216 (4.3%)	3.3	0.46* (0.40-0.54)	2,459 (4.1%)	49 (4.0%)	50.2	0.52* (0.39-0.70)	15.18* (11.01-20.93)	1.13 (0.80-1.58)
	Women	81 (0.4%)	21 (0.4%)	3.9	0.54** (0.33-0.88)	218 (0.4%)	4 (0.3%)	54.5	0.57 (0.21-1.53)	14.13* (4.71-42.42)	1.05 (0.35-3.17)
	**Subtotal**	**796 (3.7%)**	**238 (4.7%)**	**3.3**	**N/A**	**2,702 (4.5%)**	**52 (4.2%)**	**52.0**	**N/A**	**15.54* (11.39-21.19)**	**N/A**

[2] Latin American is not an ethnicity readily available in the GUMCAD and HARS datasets and was derived from the attendee's country of birth (Belize, Costa Rica, El Salvador, Guatemala, Honduras, Mexico, Nicaragua, Panama, Argentina, Bolivia, Bouvet, Brazil, Chile, Colombia, Ecuador, Falkland Islands, French Guiana, Guyana, Paraguay, Peru, South Georgia and the South Sandwich Islands, Suriname, Uruguay, Venezuela)

* p-value<0.01

** p-value<0.1

Table 3. Distribution of the number of PrEP users and PrEP need in England during the pre- (PrEP Impact Trial – October 2017 to February 2020) and post-commissioning (2021) period of PrEP by region of residence and gender.

Region	Gender	Pre-commissioning (Oct 2017 – Feb 2020)				Post-commissioning (2021)				Pre- to post-commissioning comparisons within groups	Pre- to post-commissioning comparisons across groups
		PrEP users N (%)	Non-late new HIV dx N (%)	PnR	PnR relative difference (95% CI)	PrEP users N (%)	Non-late new HIV dx N (%)	PnR	PnR relative difference (95% CI)	PnR relative difference (95% CI)	PnR relative difference (95% CI)
London	Men	10,927 (52.1%)	1,706 (34.0%)	6.4	Baseline group	32,708 (55.2%)	318 (25.8%)	102.9	Baseline group	16.06* (14.22-18.14)	Baseline group
	Women	330 (1.6%)	454 (9.0%)	0.7	0.11* (0.10-0.13)	668 (1.1%)	108 (8.8%)	6.2	0.06* (0.05-0.08)	8.51* (6.64-10.90)	0.53* (0.40-0.70)
	**Subtotal**	**11,275 (53.8%)**	**2,161 (43.1%)**	**5.2**	**N/A**	**33,569 (56.6%)**	**426 (34.5%)**	**78.8**	**N/A**	**15.10* (13.58-16.79)**	**N/A**
Midlands & East of England	Men	2,518 (12.0%)	737 (14.7%)	3.4	0.53* (0.48-0.59)	5,816 (9.8%)	218 (17.7%)	26.7	0.26* (0.22-0.31)	7.81* (6.67-9.15)	0.49* (0.40-0.59)
	Women	92 (0.4%)	334 (6.7%)	0.3	0.04* (0.03-0.05)	152 (0.3%)	96 (7.8%)	1.6	0.02* (0.01-0.02)	5.75* (4.07-8.11)	0.36* (0.25-0.52)
	**Subtotal**	**2,615 (12.5%)**	**1,072 (21.4%)**	**2.4**	**N/A**	**6,726 (11.3%)**	**314 (25.4%)**	**21.4**	**N/A**	**8.78* (7.68-10.04)**	**N/A**
North of England	Men	3,274 (15.6%)	783 (15.6%)	4.2	0.65* (0.59-0.72)	9,231 (15.6%)	207 (16.8%)	44.6	0.43* (0.36-0.52)	10.67* (9.10-12.49)	0.66* (0.54-0.81)
	Women	84 (0.4%)	233 (4.6%)	0.4	0.06* (0.04-0.07)	180 (0.3%)	78 (6.3%)	2.3	0.02* (0.02-0.03)	6.40* (4.45-9.22)	0.40* (0.27-0.59)
	**Subtotal**	**3,366 (16.1%)**	**1,016 (20.2%)**	**3.3**	**N/A**	**9,858 (16.6%)**	**285 (23.1%)**	**34.6**	**N/A**	**10.44* (9.10-11.97)**	**N/A**
South of England	Men	3,595 (17.2%)	571 (11.4%)	6.3	0.98 (0.89-1.09)	8,454 (14.3%)	152 (12.3%)	55.6	0.54* (0.45-0.66)	8.83* (7.36-10.61)	0.55* (0.44-0.69)
	Women	94 (0.4%)	198 (3.9%)	0.5	0.07* (0.06-0.10)	179 (0.3%)	56 (4.5%)	3.2	0.03* (0.02-0.04)	6.73* (4.57-9.92)	0.42* (0.28-0.63)
	**Subtotal**	**3,700 (17.7%)**	**770 (15.3%)**	**4.8**	**N/A**	**9,147 (15.4%)**	**209 (16.9%)**	**43.8**	**N/A**	**9.11* (7.78-10.66)**	**N/A**

*p-value<0.01

Table 4. Distribution of the number of PrEP users and PrEP need in England during the pre- (PrEP Impact Trial – October 2017 to February 2020) and post-commissioning (2021) period of PrEP by region of residence and ethnicity.

Region	Ethnicity	Pre-commissioning (Oct 2017 - Feb 2020)				Post-commissioning (2021)				Pre- to post-commissioning comparisons within groups	Pre- to post-commissioning comparisons across groups
		PrEP users (%)	Non-late new HIV diagnoses (%)	PnR	PnR ratio (95% CI)	PrEP users (%)	Non-late new HIV diagnoses (%)	PnR	PnR ratio (95% CI)	PnR ratio (95% CI)	PnR ratio (95% CI)
London	White	7,655 (36.5%)	883 (17.6%)	8.67	Baseline group	22,371 (37.7%)	134 (10.9%)	166.95	Baseline group	19.26* (16.03-23.14)	Baseline group
	Black African	261 (1.2%)	282 (5.6%)	0.93	0.11* (0.09-0.13)	843 (1.4%)	64 (5.2%)	13.17	0.08* (0.06-0.11)	14.23 (10.49-19.30)	0.74** (0.52-1.05)
	Black other[3]	385 (1.8%)	138 (2.7%)	2.79	0.32* (0.26-0.40)	1,032 (1.7%)	36 (2.9%)	28.67	0.17* (0.12-0.25)	10.28* (6.99-15.10)	0.53* (0.35-0.82)
	Asian	702 (3.3%)	138 (2.7%)	5.09	0.59* (0.48-0.71)	3,145 (5.3%)	33 (2.7%)	95.30	0.57* (0.39-0.84)	18.73* (12.70-27.63)	0.97 (0.63-1.50)
	Mixed/other	1,253 (6.0%)	311 (6.2%)	4.03	0.46* (0.40-0.54)	3,594 (6.1%)	43 (3.5%)	83.58	0.50* (0.35-0.71)	20.75* (14.98-28.72)	1.08 (0.74-1.57)
	Not stated	1,019 (4.9%)	409 (8.1%)	2.49	0.29* (0.25-0.33)	2,584 (4.4%)	116 (9.4%)	22.28	0.13* (0.10-0.17)	8.94* (7.19-11.12)	0.46* (0.35-0.62)
	**Subtotal**	**11,275 (53.8%)**	**2,161 (43.1%)**	**5.22**	**N/A**	**33,569 (56.6%)**	**426 (34.5%)**	**78.80**	**N/A**	**15.10* (13.58-16.79)**	**N/A**
Midlands & East of England	White	2,138 (10.2%)	554 (11.0%)	3.86	0.45* (0.40-0.50)	5,285 (8.9%)	132 (10.7%)	40.04	0.24* (0.19-0.31)	10.37* (8.52-12.63)	0.54* (0.41-0.70)
	Black African	37 (0.2%)	194 (3.9%)	0.19	0.02* (0.02-0.03)	99 (0.2%)	63 (5.1%)	1.57	0.01* (0.01-0.01)	8.24* (5.14-13.22)	0.43* (0.26-0.71)
	Black other	35 (0.2%)	48 (1.0%)	0.73	0.08* (0.05-0.13)	119 (0.2%)	18 (1.5%)	6.61	0.04* (0.02-0.07)	9.07* (4.69-17.54)	0.47** (0.24-0.93)
	Asian	154 (0.7%)	73 (1.5%)	2.11	0.24* (0.18-0.32)	478 (0.8%)	26 (2.1%)	18.38	0.11* (0.07-0.17)	8.71* (5.38-14.13)	0.45* (0.27-0.76)
	Mixed/other	130 (0.6%)	59 (1.2%)	2.20	0.25* (0.19-0.35)	316 (0.5%)	19 (1.5%)	16.63	0.10* (0.06-0.16)	7.55* (4.33-13.16)	0.39* (0.22-0.70)
	Not stated	121 (0.6%)	144 (2.9%)	0.84	0.10* (0.08-0.12)	429 (0.7%)	56 (4.5%)	7.66	0.05* (0.03-0.06)	9.12* (6.31-13.18)	0.47* (0.31-0.71)
	**Subtotal**	**2,615 (12.5%)**	**1,072 (21.4%)**	**2.44**	**N/A**	**6,726 (11.3%)**	**314 (25.4%)**	**21.42**	**N/A**	**8.78* (7.68-10.04)**	**N/A**
North of England	White	2,958 (14.1%)	645 (12.9%)	4.59	0.53* (0.47-0.59)	8,034 (13.5%)	149 (12.1%)	53.92	0.32* (0.26-0.41)	11.76* (9.79-14.12)	0.61* (0.47-0.79)
	Black African	34 (0.2%)	125 (2.5%)	0.27	0.03* (0.02-0.05)	102 (0.2%)	40 (3.2%)	2.55	0.02* (0.01-0.02)	9.38* (5.54-15.88)	0.49** (0.28-0.85)
	Black other	24 (0.1%)	24 (0.5%)	1.00	0.12* (0.07-0.20)	107 (0.2%)	7 (0.6%)	15.29	0.09* (0.04-0.20)	15.29* (5.90-39.57)	0.79 (0.30-2.09)
	Asian	108 (0.5%)	40 (0.8%)	2.70	0.31* (0.22-0.45)	502 (0.8%)	11 (0.9%)	45.64	0.27* (0.15-0.51)	16.90* (8.40-34.00)	0.88 (0.43-1.81)
	Mixed/other	164 (0.8%)	72 (1.4%)	2.28	0.26* (0.20-0.35)	503 (0.8%)	27 (2.2%)	18.63	0.11* (0.07-0.17)	8.18* (5.08-13.17)	0.42* (0.25-0.71)
	Not stated	78 (0.4%)	110 (2.2%)	0.71	0.08* (0.06-0.11)	610 (1.0%)	51 (4.1%)	11.96	0.07* (0.05-0.10)	16.87* (11.23-25.34)	0.88 (0.56-1.37)
	**Subtotal**	**3,366 (16.1%)**	**1,016 (20.2%)**	**3.31**	**N/A**	**9,858 (16.6%)**	**285 (23.1%)**	**34.59**	**N/A**	**10.44* (9.10-11.97)**	**N/A**
South of England	White	3,138 (15.0%)	482 (9.6%)	6.51	0.75* (0.67-0.85)	7,646 (12.9%)	111 (9.0%)	68.88	0.41* (0.32-0.53)	10.58* (8.57-13.06)	0.55* (0.42-0.73)
	Black African	42 (0.2%)	87 (1.7%)	0.48	0.06* (0.04-0.08)	69 (0.1%)	34 (2.8%)	2.03	0.01* (0.01-0.02)	4.20* (2.42-7.30)	0.22* (0.12-0.39)
	Black other	37 (0.2%)	23 (0.5%)	1.61	0.19* (0.11-0.31)	80 (0.1%)	11 (0.9%)	7.27	0.04* (0.02-0.08)	4.52* (2.00-10.24)	0.23* (0.10-0.54)
	Asian	118 (0.6%)	41 (0.8%)	2.88	0.33* (0.23-0.48)	403 (0.7%)	12 (1.0%)	33.58	0.20* (0.11-0.37)	11.67* (5.94-22.92)	0.61 (0.30-1.22)
	Mixed/other	173 (0.8%)	55 (1.1%)	3.15	0.36* (0.27-0.50)	463 (0.8%)	12 (1.0%)	38.58	0.23* (0.13-0.42)	12.27* (6.41-23.46)	0.64 (0.32-1.25)
	Not stated	192 (0.9%)	82 (1.6%)	2.34	0.27* (0.21-0.35)	486 (0.8%)	29 (2.4%)	16.76	0.10* (0.07-0.15)	7.16* (4.54-11.28)	0.37* (0.23-0.61)
	**Subtotal**	**3,700 (17.7%)**	**770 (15.3%)**	**4.81**	**N/A**	**9,147 (15.4%)**	**209 (16.9%)**	**43.77**	**N/A**	**9.11* (7.78-10.66)**	**N/A**

[3] Here Black other includes people of Black Caribbean ethnicity.

* p-value<0.01

** p-value<0.1

Table 5. Distribution of the number of PrEP users and PrEP need in England during the pre- (PrEP Impact Trial – October 2017 to February 2020) and post-commissioning (2021) period of PrEP by Index of Multiple Deprivation (IMD) and gender

IMD	Gender	Pre-commissioning (Oct 2017 – Feb 2020)				Pre-commissioning (Oct 2017 – Feb 2020)				Pre- to post-commissioning comparisons within groups	Pre- to post-commissioning comparisons across groups
		PrEP users N (%)	Non-late new HIV dx N (%)	PnR	PnR relative difference (95% CI)	PrEP users N (%)	Non-late new HIV dx N (%)	PnR	PnR relative difference (95% CI)	PnR relative difference (95% CI)	PnR relative difference (95% CI)
5th Quintile - least deprived	Men	1,973 (9.3%)	294 (5.9%)	6.71	Baseline group	5,254 (8.7%)	50 (4.1%)	105.08	Baseline group	15.66* (11.55-21.23)	Baseline group
	Women	60 (0.3%)	72 (1.4%)	0.83	0.12* (0.09-0.18)	92 (0.2%)	20 (1.6%)	4.60	0.04* (0.03-0.08)	5.52* (3.05-9.98)	0.35* (0.18-0.69)
	**Subtotal**	**2,035 (9.6%)**	**367 (7.3%)**	**5.54**	**N/A**	**5,624 (9.3%)**	**71 (5.8%)**	**79.21**	**N/A**	**14.29* (11.02-18.51)**	**N/A**
4th Quintile	Men	2,960 (13.9%)	473 (9.4%)	6.26	0.93 (0.80-1.09)	8,354 (13.8%)	107 (8.7%)	78.07	0.74** (0.53-1.04)	12.48* (10.07-15.45)	0.80 (0.55-1.16)
	Women	85 (0.4%)	94 (1.9%)	0.90	0.13* (0.10-0.19)	204 (0.3%)	37 (3.0%)	5.51	0.05* (0.03-0.08)	6.10* (3.86-9.63)	0.39* (0.22-0.67)
	**Subtotal**	**3,051 (14.3%)**	**567 (11.3%)**	**5.38**	**N/A**	**8,925 (14.8%)**	**144 (11.7%)**	**61.98**	**N/A**	**11.52* (9.55-13.89)**	**N/A**
3rd Quintile	Men	4,443 (20.9%)	687 (13.7%)	6.47	0.96 (0.83-1.12)	12,302 (20.4%)	154 (12.5%)	79.88	0.76** (0.55-1.05)	12.35* (10.34-14.76)	0.79 (0.55-1.12)
	Women	126 (0.6%)	180 (3.6%)	0.70	0.10* (0.08-0.14)	219 (0.4%)	58 (4.7%)	3.78	0.04* (0.02-0.05)	5.39* (3.73-7.80)	0.34* (0.21-0.56)
	**Subtotal**	**4,576 (21.5%)**	**868 (17.3%)**	**5.27**	**N/A**	**12,917 (21.4%)**	**212 (17.2%)**	**60.93**	**N/A**	**11.56* (9.91-13.48)**	**N/A**
2nd Quintile	Men	6,690 (31.4%)	1,112 (22.2%)	6.02	0.90 (0.78-1.03)	18,768 (31.1%)	225 (18.2%)	83.41	0.79 (0.58-1.08)	13.86* (11.98-16.04)	0.89 (0.63-1.24)
	Women	169 (0.8%)	322 (6.4%)	0.52	0.08* (0.06-0.10)	369 (0.6%)	82 (6.6%)	4.50	0.04* (0.03-0.06)	8.57* (6.33-11.61)	0.55* (0.36-0.84)
	**Subtotal**	**6,876 (32.3%)**	**1,434 (28.6%)**	**4.79**	**N/A**	**19,628 (32.5%)**	**307 (24.9%)**	**63.93**	**N/A**	**13.33* (11.75-15.13)**	**N/A**
1st Quintile - most deprived	Men	4,248 (20.0%)	933 (18.6%)	4.55	0.68* (0.59-0.78)	11,512 (19.1%)	273 (22.1%)	42.17	0.40* (0.30-0.54)	9.26* (8.06-10.65)	0.59* (0.42-0.83)
	Women	160 (0.8%)	363 (7.2%)	0.44	0.07* (0.05-0.08)	295 (0.5%)	110 (8.9%)	2.68	0.03* (0.02-0.04)	6.08* (4.57-8.11)	0.39* (0.26-0.59)
	**Subtotal**	**4,418 (20.7%)**	**1,296 (25.8%)**	**3.41**	**N/A**	**12,187 (20.2%)**	**383 (31.0%)**	**31.82**	**N/A**	**9.33* (8.29-10.51)**	**N/A**
Not reported	Men	312 (1.5%)	298 (5.9%)	1.05	0.16* (0.13-0.19)	979 (1.6%)	86 (7.0%)	11.38	0.11* (0.08-0.15)	10.87* (8.29-14.27)	0.69** (0.46-1.04)
	Women	23 (0.1%)	188 (3.7%)	0.12	0.02* (0.01-0.03)	019 (0.0%)	31 (2.5%)	0.61	0.01* (0.00-0.01)	5.01* (2.45-10.26)	0.32* (0.15-0.70)
	**Subtotal**	**336 (1.6%)**	**487 (9.7%)**	**0.69**	**N/A**	**1,103 (1.8%)**	**117 (9.5%)**	**9.43**	**N/A**	**13.66* (10.79-17.30)**	**N/A**

* p-value<0.01
** p-value<0.1
