# Supplementary material for: Impact of national commissioning of pre-exposure prophylaxis (PrEP) on equity of access in England: a PrEP-to-need ratio investigation
Source: Sex Transm Infect. 2024 Mar 20;100(3):166–72. doi: 10.1136/sextrans-2023-055989 (PMC11041607; doi:10.1136/sextrans-2023-055989)
Supplement: Supplementary data [file sextrans-2023-055989supp002.pdf]

BMJ Publishing Group Limited (BMJ) disclaims all liability and responsibility arising from any reliance placed on this supplemental material which has been supplied by the author(s)

Supplementary material 2

Table 1. Distribution of the number of PrEP users and PrEP need (via the recent new HIV diagnosis proxy) in England during the pre- (PrEP Impact Trial – October 2017 to February 2020) and post-commissioning (2021) period of PrEP by age and gender (including gender and sexual orientation minorities)

Age	Gender	Pre-commissioning (Oct 2017 – Feb 2020)				Post-commissioning (2021)				Pre- to post-commissioning comparisons within groups	Pre- to post-commissioning comparisons across groups
		PrEP users N (%)	Recent new HIV dx N (%)	PnR	PnR relative difference (95% CI)	PrEP users N (%)	Recent new HIV dx N (%)	PnR	PnR relative difference (95% CI)	PnR relative difference (95% CI)	PnR relative difference (95% CI)
Overall	**Total**	**21,292 (100.0%)**	**703 (100.0%)**	**30.29**	**N/A**	**60,384 (100.0%)**	**148 (100.0%)**	**408.00**	**N/A**	**13.47* (11.28-16.09)**	**N/A**
	Men, of which:	20,626 (96.9%)	601 (85.5%)	34.32	Baseline group	57,169 (94.7%)	122 (82.4%)	468.60	Baseline group	13.65* (11.23-16.60)	Baseline group
	MSM	20,349 (95.6%)	474 (67.4%)	42.93	N/A	49,543 (82.0%)	74 (50.0%)	669.50	N/A	15.60* (12.20-19.93)	N/A
	Heterosexual men	277 (1.3%)	127 (18.1%)	2.18	N/A	7,626 (12.6%)	48 (32.4%)	158.88	N/A	72.84* (51.17-103.68)	N/A
	Women	623 (2.9%)	102 (14.5%)	6.11	0.18* (0.14-0.22)	1,198 (2.0%)	25 (16.9%)	47.92	0.10* (0.07-0.16)	7.85* (5.01-12.28)	0.57** (0.35-0.94)
	Transgender people[1]	456 (2.1%)	9 (1.3%)	50.67	N/A	527 (0.9%)	0 (0.0%)	N/A	N/A	N/A	N/A
16 to 24	Men	2,973 (14.0%)	107 (15.2%)	27.79	Baseline group	8,691 (14.4%)	19 (12.8%)	457.42	Baseline group	16.46* (10.09-26.86)	Baseline group
	Women	115 (0.5%)	22 (3.1%)	5.23	0.19* (0.11-0.31)	261 (0.4%)	8 (5.4%)	32.63	0.07* (0.03-0.16)	6.24* (2.70-14.43)	0.38** (0.14-1.00)
	**Subtotal**	**3,104 (14.6%)**	**129 (18.3%)**	**24.06**	**N/A**	**9,313 (15.4%)**	**28 (18.9%)**	**332.61**	**N/A**	**13.82* (9.17-20.84)**	**N/A**
25 to 34	Men	8,348 (39.2%)	241 (34.3%)	34.64	1.25** (0.99-1.57)	23,603 (39.1%)	52 (35.1%)	453.90	0.99 (0.59-1.68)	13.10* (9.70-17.70)	0.80 (0.45-1.41)
	Women	251 (1.2%)	26 (3.7%)	9.65	0.35* (0.22-0.54)	492 (0.8%)	6 (4.1%)	82.00	0.18* (0.07-0.45)	8.49* (3.45-20.91)	0.52 (0.19-1.44)
	**Subtotal**	**8,617 (40.5%)**	**267 (38.0%)**	**32.27**	**N/A**	**24,830 (41.1%)**	**58 (39.2%)**	**428.10**	**N/A**	**13.26* (9.98-17.64)**	**N/A**
35 to 49	Men	6,832 (32.1%)	181 (25.7%)	37.75	1.36** (1.07-1.73)	17,972 (29.8%)	34 (23.0%)	528.59	1.16 (0.66-2.03)	14.00* (9.70-20.22)	0.85 (0.46-1.57)
	Women	182 (0.9%)	31 (4.4%)	5.87	0.21* (0.14-0.32)	348 (0.6%)	5 (3.4%)	69.60	0.15* (0.06-0.41)	11.86* (4.53-31.01)	0.72 (0.24-2.12)
	**Subtotal**	**7,021 (33.0%)**	**212 (30.2%)**	**33.12**	**N/A**	**18,889 (31.3%)**	**39 (26.4%)**	**484.33**	**N/A**	**14.62* (10.38-20.60)**	**N/A**
50 to 64	Men	2,202 (10.3%)	61 (8.7%)	36.10	1.30 (0.94-1.79)	6,184 (10.2%)	13 (8.8%)	475.69	1.04 (0.51-2.11)	13.18* (7.23-24.03)	0.80 (0.37-1.74)
	Women	64 (0.3%)	18 (2.6%)	3.56	0.13* (0.07-0.22)	88 (0.1%)	9 (6.1%)	9.78	0.02* (0.01-0.05)	2.75** (1.16-6.52)	0.17* (0.06-0.45)
	**Subtotal**	**2,267 (10.6%)**	**79 (11.2%)**	**28.70**	**N/A**	**6,583 (10.9%)**	**19 (12.8%)**	**346.47**	**N/A**	**12.07* (7.30-19.97)**	**N/A**
65 and over	Men	271 (1.3%)	11 (1.6%)	24.64	0.89 (0.47-1.67)	718 (1.2%)	4 (2.7%)	179.50	0.39** (0.13-1.16)	7.29* (2.30-23.08)	0.44 (0.13-1.55)
	Women	11 (0.1%)	5 (0.7%)	2.20	0.08* (0.03-0.23)	7 (0.0%)	0 (0.0%)	N/A	N/A	N/A	N/A
	**Subtotal**	**283 (1.3%)**	**16 (2.3%)**	**17.69**	**N/A**	**766 (1.3%)**	**4 (2.7%)**	**191.50**	**N/A**	**10.83* (3.59-32.67)**	**N/A**

[1] Transgender people includes transgender men, transgender women and those who identified as non-binary and was only available for the national gender breakdown to avoid small number masking, as required by UKHSA data request policy.
* p-value<0.01
**p-value<0.1

Table 2. Distribution of the number of PrEP users and PrEP need (via the recent new HIV diagnosis proxy) in England during the pre- (PrEP Impact Trial – October 2017 to February 2020) and post-commissioning (2021) period of PrEP by ethnicity and gender

Ethnicity	Gender	Pre-commissioning (Oct 2017 – Feb 2020)				Post-commissioning (2021)				Pre- to post-commissioning comparisons within groups	Pre- to post-commissioning comparisons across groups
		PrEP users N (%)	Recent new HIV dx N (%)	PnR	PnR relative difference (95% CI)	PrEP users N (%)	Recent new HIV dx N (%)	PnR	PnR relative difference (95% CI)	PnR relative difference (95% CI)	PnR relative difference (95% CI)
White	Men	15,669 (73.6%)	397 (56.5%)	39.47	Baseline group	41,765 (69.2%)	68 (45.9%)	614.19	Baseline group	15.56* (12.02-20.14)	Baseline group
	Women	367 (1.7%)	58 (8.3%)	6.33	0.16* (0.12-0.22)	714 (1.2%)	9 (6.1%)	79.33	0.13* (0.06-0.26)	12.54* (6.14-25.59)	0.81 (0.38-1.72)
	**Subtotal**	**16,061 (75.4%)**	**455 (64.7%)**	**35.30**	**N/A**	**44,106 (73.0%)**	**78 (52.7%)**	**565.46**	**N/A**	**16.02* (12.59-20.38)**	**N/A**
Black African	Men	339 (1.6%)	27 (3.8%)	12.56	0.32* (0.21-0.48)	1,050 (1.7%)	8 (5.4%)	131.25	0.21* (0.10-0.45)	10.45* (4.70-23.23)	0.67 (0.29-1.55)
	Women	36 (0.2%)	18 (2.6%)	2.00	0.05* (0.03-0.09)	42 (0.1%)	8 (5.4%)	5.25	0.01* (0.00-0.02)	2.63** (1.02-6.75)	0.17* (0.06-0.45)
	**Subtotal**	**376 (1.8%)**	**45 (6.4%)**	**8.36**	**N/A**	**1,131 (1.9%)**	**16 (10.8%)**	**70.69**	**N/A**	**8.46* (4.73-15.14)**	**N/A**
Black Caribbean	Men	341 (1.6%)	0 (0.0%)	N/A	N/A	964 (1.6%)	0 (0.0%)	N/A	N/A	N/A	N/A
	Women	9 (0.0%)	0 (0.0%)	N/A	N/A	19 (0.0%)	0 (0.0%)	N/A	N/A	N/A	N/A
	**Subtotal**	**350 (1.6%)**	**0 (0.0%)**	**N/A**	**N/A**	**996 (1.6%)**	**0 (0.0%)**	**N/A**	**N/A**	**N/A**	**N/A**
Black Other	Men	128 (0.6%)	18 (2.6%)	7.11	0.18* (0.11-0.30)	336 (0.6%)	4 (2.7%)	84.00	0.14* (0.05-0.38)	11.81* (3.92-35.58)	0.76 (0.24-2.36)
	Women	6 (0.0%)	8 (1.1%)	0.75	0.02* (0.01-0.06)	13 (0.0%)	4 (2.7%)	3.25	0.01* (0.00-0.02)	4.33** (0.93-20.24)	0.28 (0.06-1.33)
	**Subtotal**	**134 (0.6%)**	**26 (3.7%)**	**5.15**	**N/A**	**358 (0.6%)**	**6 (4.1%)**	**59.67**	**N/A**	**11.58* (4.66-28.75)**	**N/A**
Asian	Men	1,041 (4.9%)	46 (6.5%)	22.63	0.57* (0.42-0.78)	4,413 (7.3%)	9 (6.1%)	490.33	0.80 (0.40-1.60)	21.67* (10.57-44.41)	1.39 (0.65-2.98)
	Women	46 (0.2%)	4 (0.6%)	11.50	0.29** (0.10-0.81)	86 (0.1%)	0 (0.0%)	N/A	N/A	N/A	N/A
	**Subtotal**	**1,092 (5.1%)**	**50 (7.1%)**	**21.84**	**N/A**	**4,616 (7.6%)**	**9 (6.1%)**	**512.89**	**N/A**	**23.48* (11.51-47.90)**	**N/A**
Mixed/ other	Men	1,646 (7.7%)	64 (9.1%)	25.72	0.65* (0.50-0.85)	4,675 (7.7%)	14 (9.5%)	333.93	0.54** (0.31-0.97)	12.98* (7.26-23.21)	0.83 (0.44-1.58)
	Women	87 (0.4%)	5 (0.7%)	17.40	0.44** (0.18-1.09)	175 (0.3%)	4 (2.7%)	43.75	0.07* (0.03-0.20)	2.51 (0.66-9.60)	0.16* (0.04-0.63)
	**Subtotal**	**1,740 (8.2%)**	**69 (9.8%)**	**25.22**	**N/A**	**4,960 (8.2%)**	**17 (11.5%)**	**291.76**	**N/A**	**11.57* (6.79-19.73)**	**N/A**
Not stated	Men	1,462 (6.9%)	49 (7.0%)	29.84	0.76** (0.56-1.02)	3,966 (6.6%)	24 (16.2%)	165.25	0.27* (0.17-0.43)	5.54* (3.39-9.06)	0.36* (0.20-0.62)
	Women	72 (0.3%)	9 (1.3%)	8.00	0.20* (0.10-0.41)	149 (0.2%)	4 (2.7%)	37.25	0.06* (0.02-0.17)	4.66** (1.39-15.63)	0.30** (0.09-1.03)
	**Subtotal**	**1,539 (7.2%)**	**58 (8.3%)**	**26.53**	**N/A**	**4,217 (7.0%)**	**22 (14.9%)**	**191.68**	**N/A**	**7.22* (4.41-11.84)**	**N/A**
Latin American[2]	Men	714 (3.4%)	24 (3.4%)	29.75	0.75 (0.50-1.15)	2,459 (4.1%)	4 (2.7%)	614.75	1.00 (0.36-2.75)	20.67* (7.15-59.76)	1.33 (0.45-3.96)
	Women	81 (0.4%)	4 (0.6%)	20.25	0.51 (0.19-1.41)	218 (0.4%)	4 (2.7%)	54.50	0.09* (0.03-0.25)	2.69 (0.66-11.02)	0.17** (0.04-0.72)
	**Subtotal**	**796 (3.7%)**	**24 (3.4%)**	**33.17**	**N/A**	**2,702 (4.5%)**	**4 (2.7%)**	**675.50**	**N/A**	**20.37* (7.05-58.88)**	**N/A**

[2] Latin American is not an ethnicity readily available in the GUMCAD and HARS datasets and was derived from the attendee's country of birth (Belize, Costa Rica, El Salvador, Guatemala, Honduras, Mexico, Nicaragua, Panama, Argentina, Bolivia, Bouvet, Brazil, Chile, Colombia, Ecuador, Falkland Islands, French Guiana, Guyana, Paraguay, Peru, South Georgia and the South Sandwich Islands, Suriname, Uruguay, Venezuela)

* p-value<0.01

** p-value<0.1

Table 3. Distribution of the number of PrEP users and PrEP need (via the recent new HIV diagnosis proxy) in England during the pre- (PrEP Impact Trial – October 2017 to February 2020) and post-commissioning (2021) period of PrEP by region of residence and gender.

Region	Gender	Pre-commissioning (Oct 2017 – Feb 2020)				Post-commissioning (2021)				Pre- to post-commissioning comparisons within groups	Pre- to post-commissioning comparisons across groups
		PrEP users N (%)	Recent new HIV dx N (%)	PnR	PnR relative difference (95% CI)	PrEP users N (%)	Recent new HIV dx N (%)	PnR	PnR relative difference (95% CI)	PnR relative difference (95% CI)	PnR relative difference (95% CI)
London	Men	10,927 (52.1%)	287 (40.8%)	38.07	Baseline group	32,708 (55.2%)	48 (32.4%)	681.42	Baseline group	17.90* (13.17-24.31)	Baseline group
	Women	330 (1.6%)	34 (4.8%)	9.71	0.25* (0.18-0.37)	668 (1.1%)	8 (5.4%)	83.50	0.12* (0.06-0.26)	8.60* (3.94-18.79)	0.48** (0.21-1.11)
	**Subtotal**	**11,275 (53.8%)**	**321 (45.7%)**	**35.12**	**N/A**	**33,569 (56.6%)**	56 (37.8%)	**599.45**	**N/A**	**17.07* (12.84-22.69)**	**N/A**
Midlands & East of England	Men	2,518 (12.0%)	97 (13.8%)	25.96	0.68* (0.54-0.86)	5,816 (9.8%)	27 (18.2%)	215.41	0.32* (0.20-0.51)	8.30* (5.40-12.74)	0.46* (0.27-0.79)
	Women	92 (0.4%)	26 (3.7%)	3.54	0.09* (0.06-0.15)	152 (0.3%)	7 (4.7%)	21.71	0.03* (0.01-0.07)	6.14* (2.56-14.70)	0.34** (0.14-0.87)
	**Subtotal**	**2,615 (12.5%)**	**123 (17.5%)**	**21.26**	**N/A**	**6,726 (11.3%)**	34 (23.0%)	**197.82**	**N/A**	**9.30* (6.35-13.64)**	**N/A**
North of England	Men	3,274 (15.6%)	139 (19.8%)	23.55	0.62* (0.50-0.76)	9,231 (15.6%)	27 (18.2%)	341.89	0.50* (0.31-0.80)	14.52* (9.59-21.96)	0.81 (0.48-1.36)
	Women	84 (0.4%)	23 (3.3%)	3.65	0.10* (0.06-0.15)	180 (0.3%)	9 (6.1%)	20.00	0.03* (0.01-0.06)	5.48* (2.43-12.35)	0.31* (0.13-0.73)
	**Subtotal**	**3,366 (16.1%)**	**162 (23.0%)**	**20.78**	**N/A**	**9,858 (16.6%)**	36 (24.3%)	**273.83**	**N/A**	**13.18* (9.16-18.95)**	**N/A**
South of England	Men	3,595 (17.2%)	78 (11.1%)	46.09	1.21 (0.94-1.56)	8,454 (14.3%)	24 (16.2%)	352.25	0.52* (0.32-0.84)	7.64* (4.83-12.10)	0.43* (0.25-0.74)
	Women	94 (0.4%)	19 (2.7%)	4.95	0.13* (0.08-0.22)	179 (0.3%)	4 (2.7%)	44.75	0.07* (0.02-0.18)	9.05* (2.99-27.36)	0.51 (0.16-1.59)
	**Subtotal**	**3,700 (17.7%)**	**97 (13.8%)**	**38.14**	**N/A**	**9,147 (15.4%)**	22 (14.9%)	**415.77**	**N/A**	**10.90* (6.85-17.34)**	**N/A**

* p-value<0.01
** p-value<0.1
