# Supplementary material for: Impact of national commissioning of pre-exposure prophylaxis (PrEP) on equity of access in England: a PrEP-to-need ratio investigation
Source: Sex Transm Infect. 2024 Mar 20;100(3):166–72. doi: 10.1136/sextrans-2023-055989 (PMC11041607; doi:10.1136/sextrans-2023-055989)
Supplement: Supplementary data [file sextrans-2023-055989supp003.pdf]

Supplementary material 3

Table 1. Distribution of the number of PrEP users and PrEP need as defined by UKHSA (1) in England during the post-commissioning (2021) period of PrEP by age and gender (including gender and sexual orientation minorities)

Age	Gender	Post-commissioning (2021)			
		PrEP users (N, %)	SSHS patients with PrEP need (N, %)	PnR	PnR relative difference (95% CI)
Overall	**Total**	**60,384 (100.0%)**	**87,828 (100.0%)**	**0.69**	**N/A**
	Men, of which:	57,169 (94.7%)	80,877 (92.1%)	0.71	Baseline group
	MSM	49,543 (82.0%)	70,076 (79.8%)	0.71	N/A
	Heterosexual men	7,626 (12.6%)	10,801 (12.3%)	0.71	N/A
	Women	1,198 (2.0%)	3,901 (4.4%)	0.31	0.43* (0.41-0.46)
	Transgender people[1]	527 (0.9%)	847 (1.0%)	0.62	N/A
16 to 24	Men	8,691 (14.4%)	14,252 (16.2%)	0.61	Baseline group
	Women	261 (0.4%)	1,159 (1.3%)	0.23	0.37* (0.32-0.42)
	**Subtotal**	**9,313 (15.4%)**	**16,088 (18.3%)**	**0.58**	**N/A**
25 to 34	Men	23,603 (39.1%)	33,354 (38.0%)	0.71	1.16* (1.12-1.20)
	Women	492 (0.8%)	1,477 (1.7%)	0.33	0.55* (0.49-0.61)
	**Subtotal**	**24,830 (41.1%)**	**35,956 (41.0%)**	**0.69**	**N/A**
35 to 49	Men	17,972 (29.8%)	24,011 (27.3%)	0.75	1.23* (1.19-1.27)
	Women	348 (0.6%)	995 (1.1%)	0.35	0.57* (0.51-0.65)
	**Subtotal**	**18,889 (31.3%)**	**25,800 (29.4%)**	**0.73**	**N/A**
50 to 64	Men	6,184 (10.2%)	8,180 (9.3%)	0.76	1.24* (1.19-1.29)
	Women	88 (0.1%)	225 (0.3%)	0.39	0.64* (0.50-0.82)
	**Subtotal**	**6,583 (10.9%)**	**8,793 (10.0%)**	**0.75**	**N/A**
65 and over	Men	718 (1.2%)	1,072 (1.2%)	0.67	1.10** (1.00-1.21)
	Women	7 (0.0%)	26 (0.0%)	0.27	0.44** (0.19-1.02)
	**Subtotal**	**766 (1.3%)**	**1,161 (1.3%)**	**0.66**	**N/A**

[1] Transgender people includes transgender men, transgender women and those who identified as non-binary and was only available for the national gender breakdown to avoid small number masking, as required by UKHSA data request policy.
* p-value<0.01
**p-value<0.1

Table 2. Distribution of the number of PrEP users and PrEP need as defined by UKHSA (1) in England during the post-commissioning (2021) period of PrEP by ethnicity and gender

Ethnicity	Gender	Post-commissioning (2021)			
		**PrEP users (N, %)**	**SSHS patients with PrEP need (N, %)**	**PnR**	**PnR relative difference (95% CI)**
White	Men	41,765 (69.2%)	58,431 (66.5%)	0.71	Baseline group
	Women	714 (1.2%)	2,382 (2.7%)	0.30	0.42* (0.39-0.46)
	**Subtotal**	**44,106 (73.0%)**	**63,190 (71.9%)**	**0.70**	**N/A**
Black African	Men	1,050 (1.7%)	1,768 (2.0%)	0.59	0.83* (0.77-0.90)
	Women	42 (0.1%)	278 (0.3%)	0.15	0.21* (0.15-0.29)
	**Subtotal**	**1,131 (1.9%)**	**2,130 (2.4%)**	**0.53**	**N/A**
Black Caribbean	Men	964 (1.6%)	1,562 (1.8%)	0.62	0.86* (0.80-0.94)
	Women	19 (0.0%)	82 (0.1%)	0.23	0.32* (0.20-0.53)
	**Subtotal**	**996 (1.6%)**	**1,663 (1.9%)**	**0.60**	**N/A**
Black Other	Men	336 (0.6%)	538 (0.6%)	0.62	0.87** (0.76-1.00)
	Women	13 (0.0%)	55 (0.1%)	0.24	0.33* (0.18-0.61)
	**Subtotal**	**358 (0.6%)**	**605 (0.7%)**	**0.59**	**N/A**
Asian	Men	4,413 (7.3%)	6,033 (6.9%)	0.73	1.02 (0.98-1.07)
	Women	86 (0.1%)	208 (0.2%)	0.41	0.58* (0.45-0.74)
	**Subtotal**	**4,616 (7.6%)**	**6,425 (7.3%)**	**0.72**	**N/A**
Mixed/ other	Men	4,675 (7.7%)	6,428 (7.3%)	0.73	1.02 (0.98-1.06)
	Women	175 (0.3%)	377 (0.4%)	0.46	0.65* (0.54-0.78)
	**Subtotal**	**4,960 (8.2%)**	**6,973 (7.9%)**	**0.71**	**N/A**
Not stated	Men	3,966 (6.6%)	6,117 (7.0%)	0.65	0.91* (0.87-0.95)
	Women	149 (0.2%)	519 (0.6%)	0.29	0.40* (0.33-0.48)
	**Subtotal**	**4,217 (7.0%)**	**6,842 (7.8%)**	**0.62**	**N/A**
Latin American flag[2]	Men	2,459 (4.1%)	3,032 (3.5%)	0.81	1.13* (1.07-1.20)
	Women	218 (0.4%)	325 (0.4%)	0.67	0.94 (0.79-1.11)
	**Subtotal**	**2,702 (4.5%)**	**3,398 (3.9%)**	**0.80**	**N/A**

[2] Latin American is not an ethnicity readily available in the GUMCAD and HARS datasets and was derived from the attendee's country of birth (Belize, Costa Rica, El Salvador, Guatemala, Honduras, Mexico, Nicaragua, Panama, Argentina, Bolivia, Bouvet, Brazil, Chile, Colombia, Ecuador, Falkland Islands, French Guiana, Guyana, Paraguay, Peru, South Georgia and the South Sandwich Islands, Suriname, Uruguay, Venezuela)

* p-value<0.01

** p-value<0.1

Table 3. Distribution of the number of PrEP users and PrEP need as defined by UKHSA (1) in England during the post-commissioning (2021) period of PrEP by region of residence and gender. *p-value<0.01

Region	Gender	Post-commissioning (2021)			
		PrEP users (N, %)	SSHS patients with PrEP need (N, %)	PnR	PnR relative difference (95% CI)
London	Men	32,708 (55.2%)	40,963 (47.6%)	0.80	Baseline group
	Women	668 (1.1%)	1,524 (1.8%)	0.44	0.55* (0.50-0.60)
	**Subtotal**	**33,569 (56.6%)**	**42,774 (49.7%)**	**0.78**	**N/A**
Midlands & East of England	Men	5,816 (9.8%)	10,393 (12.1%)	0.56	0.70* (0.68-0.73)
	Women	152 (0.3%)	773 (0.9%)	0.20	0.25* (0.21-0.29)
	**Subtotal**	**6,726 (11.3%)**	**12,337 (14.3%)**	**0.55**	**N/A**
North of England	Men	9,231 (15.6%)	15,093 (17.5%)	0.61	0.77* (0.74-0.79)
	Women	180 (0.3%)	824 (1.0%)	0.22	0.27* (0.23-0.32)
	**Subtotal**	**9,858 (16.6%)**	**16,587 (19.3%)**	**0.59**	**N/A**
South of England	Men	8,454 (14.3%)	12,924 (15.0%)	0.65	0.82* (0.79-0.85)
	Women	179 (0.3%)	704 (0.8%)	0.25	0.32* (0.27-0.38)
	**Subtotal**	**9,147 (15.4%)**	**14,385 (16.7%)**	**0.64**	**N/A**

Table 4. Distribution of the number of PrEP users and PrEP need as defined by UKHSA (1) in England during the post-commissioning (2021) period of PrEP by region of residence and ethnicity. Here Black other includes people of Black Caribbean ethnicity.

Region	Ethnicity	Post-commissioning (2021)			
		**PrEP users (N, %)**	**SSHS patients with PrEP need (N, %)**	**PnR**	**PnR ratio (95% CI)**
London	White	22,371 (37.7%)	27,489 (31.9%)	0.81	Baseline group
	Black African	843 (1.4%)	1,375 (1.6%)	0.61	0.75* (0.69-0.82)
	Black other	1,032 (1.7%)	1,635 (1.9%)	0.63	0.78* (0.72-0.84)
	Asian	3,145 (5.3%)	3,988 (4.6%)	0.79	0.97 (0.92-1.02)
	Mixed/other	3,594 (6.1%)	4,682 (5.4%)	0.77	0.94** (0.90-0.99)
	Not stated	2,584 (4.4%)	3,605 (4.2%)	0.72	0.88* (0.83-0.93)
	**Subtotal**	**33,569 (56.6%)**	**42,774 (49.7%)**	**0.78**	**N/A**
Midlands & East of England	White	5,285 (8.9%)	9,261 (10.8%)	0.57	0.70* (0.68-0.73)
	Black African	99 (0.2%)	301 (0.3%)	0.33	0.40* (0.32-0.51)
	Black other	119 (0.2%)	262 (0.3%)	0.45	0.56* (0.45-0.69)
	Asian	478 (0.8%)	876 (1.0%)	0.55	0.67* (0.60-0.75)
	Mixed/other	316 (0.5%)	628 (0.7%)	0.50	0.62* (0.54-0.71)
	Not stated	429 (0.7%)	1,009 (1.2%)	0.43	0.52* (0.47-0.59)
	**Subtotal**	**6,726 (11.3%)**	**12,337 (14.3%)**	**0.55**	**N/A**
North of England	White	8,034 (13.5%)	13,400 (15.6%)	0.60	0.74* (0.71-0.76)
	Black African	102 (0.2%)	243 (0.3%)	0.42	0.52* (0.41-0.65)
	Black other	107 (0.2%)	191 (0.2%)	0.56	0.69* (0.54-0.87)
	Asian	502 (0.8%)	816 (0.9%)	0.62	0.76* (0.68-0.85)
	Mixed/other	503 (0.8%)	814 (0.9%)	0.62	0.76* (0.68-0.85)
	Not stated	610 (1.0%)	1,123 (1.3%)	0.54	0.67* (0.60-0.74)
	**Subtotal**	**9,858 (16.6%)**	**16,587 (19.3%)**	**0.59**	**N/A**
South of England	White	7,646 (12.9%)	11,846 (13.8%)	0.65	0.79* (0.77-0.82)
	Black African	69 (0.1%)	176 (0.2%)	0.39	0.48* (0.36-0.64)
	Black other	80 (0.1%)	144 (0.2%)	0.56	0.68* (0.52-0.90)
	Asian	403 (0.7%)	613 (0.7%)	0.66	0.81* (0.71-0.92)
	Mixed/other	463 (0.8%)	718 (0.8%)	0.64	0.79* (0.70-0.89)
	Not stated	486 (0.8%)	888 (1.0%)	0.55	0.67* (0.60-0.75)
	**Subtotal**	**9,147 (15.4%)**	**14,385 (16.7%)**	**0.64**	**N/A**

* p-value<0.01
** p-value<0.1

Table 5. Distribution of the number of PrEP users and PrEP need as defined by UKHSA (1) in England during the post-commissioning (2021) period of PrEP by Index of Multiple Deprivation (IMD) and gender

IMD	Gender	Post-commissioning (2021)			
		**PrEP users (N, %)**	**SSHS patients with PrEP need (N, %)**	**PnR**	**PnR relative difference (95% CI)**
5th Quintile - least deprived	Men	5,254 (8.7%)	7,798 (8.9%)	0.67	Baseline group
	Women	92 (0.2%)	347 (0.4%)	0.27	0.39* (0.31-0.50)
	**Subtotal**	**5,624 (9.3%)**	**8,569 (9.8%)**	**0.66**	**N/A**
4th Quintile	Men	8,354 (13.8%)	12,036 (13.7%)	0.69	1.03 (0.99-1.08)
	Women	204 (0.3%)	562 (0.6%)	0.36	0.54* (0.46-0.63)
	**Subtotal**	**8,925 (14.8%)**	**13,157 (15.0%)**	**0.68**	**N/A**
3rd Quintile	Men	12,302 (20.4%)	17,076 (19.4%)	0.72	1.07* (1.03-1.12)
	Women	219 (0.4%)	737 (0.8%)	0.30	0.44* (0.38-0.51)
	**Subtotal**	**12,917 (21.4%)**	**18,378 (20.9%)**	**0.70**	**N/A**
2nd Quintile	Men	18,768 (31.1%)	25,446 (29.0%)	0.74	1.09* (1.05-1.14)
	Women	369 (0.6%)	1,079 (1.2%)	0.34	0.51* (0.45-0.57)
	**Subtotal**	**19,628 (32.5%)**	**27,258 (31.0%)**	**0.72**	**N/A**
1st Quintile - most deprived	Men	11,512 (19.1%)	16,991 (19.3%)	0.68	1.01 (0.96-1.05)
	Women	295 (0.5%)	1,100 (1.3%)	0.27	0.40* (0.35-0.45)
	**Subtotal**	**12,187 (20.2%)**	**18,695 (21.3%)**	**0.65**	**N/A**
Not reported	Men	979 (1.6%)	1,530 (1.7%)	0.64	0.95 (0.87-1.04)
	Women	19 (0.0%)	76 (0.1%)	0.25	0.37* (0.22-0.61)
	**Subtotal**	**1,103 (1.8%)**	**1,771 (2.0%)**	**0.62**	**N/A**

* p-value<0.01

References

1. Routine commissioning of HIV pre-exposure prophylaxis (PrEP) in England: Monitoring and evaluation framework. London: UKHSA; 2022.
